# Advances in Diagnosis of Respiratory Virus Infections

**DOI:** 10.1155/2010/126049

**Published:** 2010-10-19

**Authors:** Michael Loeffelholz, Tasnee Chonmaitree

**Affiliations:** ^1^Department of Pathology, The University of Texas Medical Branch, 301 University Boulevard, Galveston, TX 77555-0740, USA; ^2^Department of Pediatrics, The University of Texas Medical Branch, 301 University Boulevard, Galveston, TX 77555-0371, USA

## Abstract

The diagnosis of respiratory virus infections has evolved substantially in recent years, with the emergence of new pathogens and the development of novel detection methods. While recent advances have improved the sensitivity and turn-around time of diagnostic tests for respiratory viruses, they have also raised important issues such as cost, and the clinical significance of detecting multiple viruses in a single specimen by molecular methods. This article reviews recent advances in specimen collection and detection methods for diagnosis of respiratory virus infections, and discusses the performance characteristics and limitations of these methods.

## 1. Introduction

Respiratory virus infections have a major impact on health. Acute respiratory illnesses, mostly caused by viruses, are the most common illness experienced by otherwise healthy children and adults worldwide. Upper respiratory tract infections (URIs) such as common cold are exceedingly prevalent in infants and young children and continue to be common in older children and adults. Infants and young children may have 3–8 episodes of cold per year; those who attend daycare centers may have many more episodes per year [1–4]. URI can lead to complications such as acute otitis media, asthma exacerbation, and lower respiratory tract infections (LRIs). While LRIs such as pneumonia, bronchitis, and bronchiolitis occur much less frequently, they cause higher morbidity and some mortality, thus they are associated with high impact and greater healthcare costs. Approximately one third of children develop an LRI in the first year of life; LRI incidence decreases to 5%–10% during early school year, and 5% during preadolescent to healthy adult years [5, 6]. 

Common respiratory viruses include influenza A and B, respiratory syncytial virus A and B, parainfluenza virus types 1–3, adenovirus, rhinovirus, human metapneumovirus, and coronavirus types OC43 and 229E. Less common respiratory viruses include parainfluenza virus type 4, influenza virus C, and specific types of enteroviruses. The significance of more recently discovered viruses such as human bocavirus, coronavirus NL63, and HKU1 has yet to be elucidated [7, 8].

Clinical presentations of respiratory virus infections overlap among those caused by various viruses. In addition, clinical manifestations may mimic those of diseases caused by bacteria. Therefore, antibiotics are most often used in these infections, most of them unnecessarily. Furthermore, LRIs often require hospitalization for management such as intravenous antibiotics and symptomatic and supportive treatment. Specific antiviral treatment for respiratory virus infections is only available for influenza. Respiratory viral diagnosis is an integral part of patient management. Accurate diagnosis of specific respiratory virus infection not only improves the knowledge of disease the patient has but also can affect patient management and help prevent secondary spread of the infection. Rapid viral diagnosis may result in discontinuation of unnecessary antibiotics, initiation of antiviral drug for influenza, reduction of costs related to reduction of unnecessary investigations, and shortened hospital stay [9–11]. 

This paper describes up-to-date information on laboratory methods presently available in the diagnostic virology laboratories and those upcoming for detection of respiratory viruses. 

## 2. Specimen Collection

### 2.1. Novel Swab Types

Perhaps one of the most significant advances in the detection of respiratory viruses is the recent introduction of novel swab types such as flocked swabs. Flocked swabs consist of nylon fibers perpendicular to the swab shaft. Unlike standard woven rayon swabs which trap sample material, the fibers of flocked swabs act like a brush to collect respiratory epithelial cells and efficiently release them in transport medium [12]. Studies comparing nasal flocked swabs to nasal aspirates from children showed that the sensitivity of flocked swabs was at least equivalent for the detection of a variety of respiratory viruses [13, 14]. Similar studies comparing flocked swabs to woven rayon swabs in adult patients are needed. Another type of novel swab material is polyurethane foam. A recent study demonstrated that polyurethane foam swabs of the anterior nares performed better than flocked swabs for detection of influenza viruses in children by a rapid antigen test [15].

### 2.2. Performance of Swab Specimens versus Aspirates/Washes

Traditionally, nasal washes and aspirates have been considered to be superior to swab specimens for isolation of respiratory viruses in cell culture and detection of viral antigens in immunoassays [16–18]. However, with contemporary diagnostic assays and the use of flocked swabs, this superiority may be mitigated [13, 14, 19]. In one recent study, both nasopharyngeal and nasal swabs were found to be superior to nasal washes for the detection of influenza viruses by a rapid antigen immunoassay in pediatric subjects [20]. A recently advocated sampling approach is the swabbing of both the throat and anterior nares as a single specimen. A recent study showed excellent sensitivity of PCR for influenza virus and RSV performed on combined nose-throat specimens [21].

### 2.3. Patient/Parent-Collected Swabs

Parent-collected respiratory swabs have been utilized in research studies, where they have been shown to yield sensitivity equivalent to that of healthcare worker-collected swabs when combined with PCR testing [22]. A practical limitation to parent- or patient-collected swabs is the availability of viral transport medium. However, a recent study showed that viral RNA was stable on dry respiratory swabs, and that these swabs were more sensitive than traditionally collected respiratory specimens for detection of viruses by nucleic acid sequence-based amplification (NASBA) [23].

## 3. Laboratory Diagnosis

### 3.1. Antigen Detection Tests

#### 3.1.1. Rapid Immunoassays

Providing a result at the point of care in less than 30 minutes, rapid immunoassays are widely used for the detection of influenza viruses and RSV. Rapid immunoassay formats include membrane-based enzyme immunoassay, lateral flow immunochromatography, and optical immunoassay. Most test results are read manually by eye although one test includes an automated reader. The sensitivity of rapid RSV immunoassays in pediatric patients is generally higher than that of cell culture due to the lability of RSV [24]. The sensitivity of rapid immunoassays also depends on the study population; children often shed respiratory viruses at higher titers [25] and for longer time periods than do adults [26]. In spite of the poor sensitivity of these tests for detection of influenza virus, positive results in the emergency setting or from hospitalized patients can significantly impact patient management [27, 28]. Recent evaluations of rapid immunoassays for detection of the novel 2009 influenza A (H1N1) virus have demonstrated variable and generally poor sensitivity, in some cases 25% or lower compared to reverse transcriptase (RT)-PCR [29–31]. These data highlight the limitations of diagnostic tests that target infectious agents such as influenza A virus, that have the capability to evolve significantly and rapidly.

#### 3.1.2. Direct Fluorescent Antibody Tests

Direct fluorescent antibody (DFA) staining of viral antigens in patient specimens is generally considered to be more sensitive than rapid immunoassays [32]. The specificity of DFA is high but depends on experienced technologists. Food and Drug Administration- (FDA-) cleared commercial DFA reagents have long been available for detection and identification of influenza A and B viruses, RSV, parainfluenza viruses 1–3, and adenovirus. Recent advances in DFA tests include the availability of FDA-cleared commercial DFA reagents for detection of human metapneumovirus. A study showed excellent sensitivity (95% versus RT-PCR) of a human metapneumovirus DFA performed on nasopharyngeal aspirates from children [33]. DFA was substantially more sensitive than rapid immunoassays for detection of the novel 2009 influenza A (H1N1) virus, though less sensitive than virus isolation and/or RT-PCR [31, 34].

### 3.2. Virus Isolation

Long considered the gold standard for detection of respiratory viruses, the turn-around time of conventional cell culture in tubes for respiratory viruses is generally too long to be clinically relevant [35]. When performed on fresh specimens maintained at refrigerated temperature, virus isolation has excellent sensitivity for most respiratory viruses. The use of centrifugation-enhanced (shell vial) culture and mixed cell lines in the same vial has decreased turn-around time to 24–48 hours and streamlined workflow [36]. A study showed that shell vials containing mixed cell lines had similar sensitivity as conventional tube culture for detection of influenza and parainfluenza viruses. For RSV detection, the shell vial system was more sensitive than conventional culture, but less sensitive than a direct antigen test [37]. Virus isolation in shell vials was highly sensitive for detection of 2009 influenza A (H1N1) [31].

### 3.3. Serology

The diagnostic utility of serology generally is limited by the need to collect both acute and convalescent sera to identify either seroconversion or a fourfold rise in antibody titer. As such, serologic methods that detect IgG responses usually have little impact on patient management. Tests that detect IgM antibodies can detect acute infection, but sensitivity is reduced because serum IgM levels are often low due to repeated exposure to vaccine or circulating virus. Recent advances in serologic assays for respiratory viruses are largely limited to novel microagglutination assays that detect and measure antibody titers to novel influenza A virus subtypes. These assays utilize engineered reporter viruses—a lentiviral vector pseudotyped to contain the influenza hemagglutinin protein that eliminates the need for enhanced biocontainment facilities [38]. The pseudotyped lentiviral particles express the hemagglutinin protein but are replication deficient. Microneutralization assays employing either infectious or noninfectious reporter viruses are performed primarily for retrospective studies, epidemiologic surveys, and vaccine trials and have limited implications for routine diagnostics.

### 3.4. Nucleic Acid Amplification Tests

Nucleic acid amplification methods such as RT-PCR and nucleic acid sequence-based amplification (NASBA) are becoming more commonly used for detection of influenza virus and other respiratory viruses. Using realtime, fluorescent detection of amplified product, laboratories are able to perform molecular tests in less than 3 hours. High specificity requires judicious selection of primers and probes, optimization of amplification conditions, and interpretation of results. Continuous adherence to laboratory protocol is essential to avoid false positives due to carryover contamination. Due to the limited number and cost of FDA-cleared nucleic acid amplification tests, many laboratories perform tests developed and verified in house (referred to as laboratory-developed tests (LDTs)). LDTs (and laboratory-verified commercial kits) are viable options for the detection of common respiratory viruses such as RSV [39], as well as viruses for which no FDA-cleared commercial assays are available such as human bocavirus [40]. The development and verification of LDTs require considerable resources and expertise. As well, the performance characteristics of LDTs may vary significantly between laboratories. Consensus recommendations on verification and quality assurance of laboratory tests, including LDTs, are available [41].

#### 3.4.1. Real-Time PCR

In real-time PCR, amplification (as detected by an increase in fluorescence) and analysis occur simultaneously. Real-time PCR is well suited for quantitative analysis, but currently available commercially kits for detection of respiratory virus nucleic acids have only qualitative claims. Most real-time PCR instruments are limited in the number of emission channels available for multiplexing. As a result, commercially available multiplex real-time PCR kits are generally limited to the detection of four or fewer nucleic acid targets. One of the targets is usually an internal control. FDA-cleared real-time RT-PCR assays are currently available for the detection of influenza A (including subtypes) and influenza B viruses, RSV, parainfluenza viruses 1, 2, and 3, and human metapneumovirus. Studies have demonstrated that molecular amplifications methods including real-time RT-PCR provide the most sensitive detection of respiratory viruses [42, 43].

#### 3.4.2. Conventional PCR

Conventional PCR requires the postamplification detection of PCR product (e.g., via sequence-specific DNA probes, or electrophoresis). Either format allows for highly multiplexed assays that are able to detect more pathogens in a single PCR reaction than real-time PCR. An FDA-cleared assay utilizing conventional RT-PCR and a microarray of sequence-specific beads can detect RSV, influenza A (including subtypes) and influenza B viruses, parainfluenza viruses 1, 2, and 3, human metapneumovirus, rhinoviruses, and adenoviruses from a single RT-PCR reaction. This PCR assay format provides sensitive detection of influenza viruses, as well as the ability to detect a broad scope of respiratory pathogens [31].

The high sensitivity of molecular amplification tests has made the interpretation of positive results challenging. These assays detect respiratory virus nucleic acid in the absence of symptoms [44–46] and also indicate that nucleic acids persist following infection [47, 48]. In addition, these methods frequently detect multiple pathogens in the same specimen [47]. Additional studies are needed to fully understand the clinical significance of these data, as well as the etiologic role of newly discovered agents such as human bocavirus. Quantitative real-time PCR methods have proven useful in this regard [49].

## 4. Summary

During the past several years, we have witnessed an explosion of improvements in respiratory virus diagnostics, from novel specimen collection instruments to highly sensitive and multiplexed nucleic acid amplification tests. With the expanding list of antigen and molecular-based tests, it is now possible for laboratories to offer comprehensive testing for respiratory viruses without even performing virus isolation. However, cell culture still remains the gold standard in many laboratories. Recent advances have also been made to this traditional approach to improve the turn-around time by using a combination of shell vial cultures and immunostaining.

Because of the sensitivity and rapid turn-around time, nucleic acid amplification technologies such as PCR will likely be the focus of further advances in respiratory virus diagnostics. The future will likely promise additional commercial test kits for molecular detection of respiratory viruses, including multiplexed assays. Because of their exquisite sensitivity, nucleic acid amplification tests can create diagnostic conundrums. Additional research is needed to elucidate the clinical significance of positive PCR results that are persistent in a patient, and when two or more respiratory viruses are detected in a single specimen. 

Future research on respiratory virus diagnostics should aim towards the ability to accurately detect a spectrum of clinically significant viruses rapidly enough to affect patient management and initiation of infection control measures while keeping the costs affordable. Commercial kits provide standardization not achievable with laboratory-developed tests, but at a substantially higher cost. Ultimately, the utilization of molecular testing, particularly highly multiplexed tests in routine patient management will depend on the cost/benefit ratio. 

Current and future diagnostic options will include antigen, molecular, and culture-based methods. The performance characteristics and limitations of these methods will vary greatly with the new generations of assays. It is important that diagnostic virologists and clinicians understand these characteristics and limitations.
